# Effective Collaboration for Scaling Up Health Technologies: A Case Study of the Chlorhexidine for Umbilical Cord Care Experience

**DOI:** 10.9745/GHSP-D-17-00380

**Published:** 2018-03-21

**Authors:** Patricia S Coffey, Steve Hodgins, Amie Bishop

**Affiliations:** a PATH, Seattle, WA, USA.; b Save the Children, Washington, DC, USA. Now with School of Public, University of Alberta, Edmonton, Canada.; c Consultant, Global Health and Human Rights, Seattle, WA, USA.

## Abstract

The global health field is replete with examples of cross-organizational collaborative partnerships, such as networks, alliances, coalitions, task forces, and working groups, often established to tackle a shared global health concern, condition, or threat affecting low-income countries or communities. The purpose of this article is to review factors influencing the effectiveness of a multi-agency global health collaborative effort using the Chlorhexidine Working Group (CWG) as our case study. The CWG was established to accelerate the introduction and global scale-up of chlorhexidine for umbilical cord care to reduce infection-related neonatal morbidity and mortality in low-income countries. Questions included: how current and past CWG members characterized the effectiveness, productivity, collaboration, and leadership of the CWG; what factors facilitated or hindered group function; institutional or individual reasons for participating and length of participation in the CWG; and lessons that might be relevant for future global collaborative partnerships. Data were collected through in-depth, semistructured individual interviews with 19 group members and a review of key guiding documents. Six domains of internal coalition functioning (leadership, interpersonal relationships, task focus, participant benefits and costs, sustainability planning, and community support) were used to frame and describe the functioning of the CWG. Collaboration effectiveness was found to depend on: (1) leadership that maintained a careful balance between discipline and flexibility, (2) a strong secretariat structure that supported the evolution of trust and transparent communication in interpersonal relationships, (3) shared goals that allowed for task focus, (4) diverse membership and active involvement from country-level participants, which created a positive benefit-cost ratio for participants, (5) sufficient resources to support the partnership and build sustainable capacity for members to accelerate the transfer of knowledge, and (6) support from the global health community across multiple organizations. Successful introduction and scale-up of new health interventions require effective collaboration across multiple organizations and disciplines, at both global and country levels. The participatory collaborative partnership approach utilized by the Chlorhexidine Working Group offers an instructive learning case.

## Abstract (precis)

Facilitating factors for the Chlorhexidine Working Group: (1) strong, transparent leadership by a neutral broker, promoting shared ownership among all members; (2) reliable internal and external communication; (3) well-defined terms of reference building on common interest around a simple, effective health intervention; (4) clear benefits of participation, including access to evidence and technical assistance; and (5) adequate resources to support the secretariat functions.

## INTRODUCTION

The global health field is replete with examples of cross-organizational collaborative partnerships, such as networks, alliances, coalitions, task forces, and working groups, often established to tackle a shared global health concern, condition, or threat affecting low-income countries or communities. These efforts generally bring together expertise drawn from a range of entities such as NGOs, universities, donors, private-sector companies, government, United Nations (UN) agencies, and communities. Common goals of these collaborative efforts include resource mobilization, policy change, knowledge generation and dissemination, research, creation of new technologies/approaches, or the introduction and scale up of evidence-based interventions.

A variety of disciplines, such as sociology, public policy, economics, and political science, have examined the effectiveness and impact of collaborative partnerships and collective action. The examination of whether, how, and why coalitions work has employed social network theory,^1^^,^^2^ relational theories of coordination,^3^^–^^5^ group dynamics/teaming,^6^ and organizational development theory.^7^^,^^8^ In public health, there is a well-developed literature on community coalitions. Zakocs et al.^9^ found that factors that enhance community coalition effectiveness include having formal governance procedures, encouraging strong leadership, fostering active participation, and cultivating diverse membership. Other investigators have highlighted the importance of balancing the autonomy of individual members with the need for collective action and accountability. This balance can be accomplished by creating a shared culture and mindset that allows for members to both protect and advance the interests of their own organizations and those of the partnership as a whole. Essential to success is the need to be “relentlessly explicit about values, principles, and practices”^10^ so that tensions over visibility and credit attribution can be superseded by a shared recognition of the need to focus on common purpose. Because collaborative networks bring disparate groups together to work toward a common cause, the role of individuals who facilitate the flow of information between such groups is key. These individuals, known as “bridges,” “brokers,” or “boundary spanners,”^11^ must be seen as trustworthy intermediaries to be effective in closing gaps between perspectives and thus increasing understanding, cooperation, and information sharing across groups.

The purpose of this article is to investigate factors affecting the effectiveness of a multi-agency collaborative effort, as perceived by participants, using the Chlorhexidine Working Group (CWG) global collaboration as our case study. Although ad hoc collaboration around this topic had been functioning since 2002, the CWG was formally established in 2012 to accelerate the introduction and global scale up of chlorhexidine for umbilical cord care to reduce infection-related neonatal mortality and morbidity in low-income countries.

The purpose of this article is to investigate factors affecting the effectiveness of a multi-agency global collaboration.

## METHODS

### Theoretical Model

**FIGURE 1.**
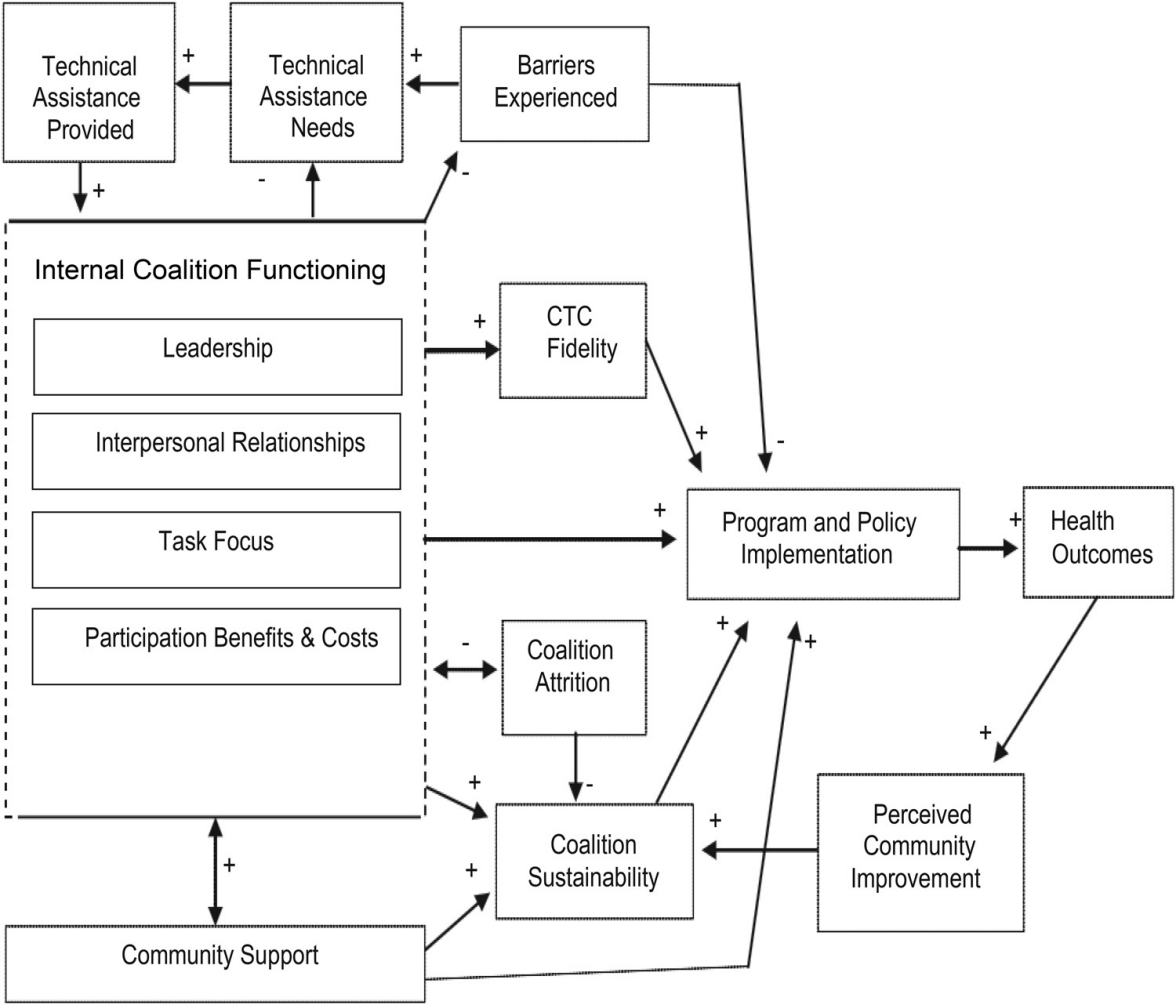
Theoretical Model of Coalition Functioning Abbreviation: CTC, Communities That Care. Source: Brown, Feinberg, and Greenberg.^12^

The theoretical model of coalition functioning developed by Brown and colleagues serves as a helpful framework for investigating the experience of the CWG.^12^ In this model (Figure 1), health outcomes are mediated by program or policy implementation, which is supported by effective collaborative partnerships. We selected this model because it aligns with the strategy of the CWG, and the domains in the framework have been empirically validated and can be used to measure the relative effectiveness of a successful collaborative effort to implement a new program or policy.

In this model, the 6 domains of internal coalition functioning that may affect the effectiveness of program implementation in a collaborative effort are:

1. Leadership
2. Interpersonal relationships
3. Task focus
4. Participant benefits and costs
5. Sustainability planning
6. Community support

6 domains of internal coalition functioning may affect the effectiveness of collaborative implementation efforts: leadership, interpersonal relationships, task focus, benefits and costs, sustainability planning, and community support.

In this model, leadership is vital to creating a collective force that can achieve common goals. Interpersonal relationships promote trust and commitment and are the pathways that allow effective collaboration to occur. Task focus is important because it maintains focus on the issues at hand and minimizes peripheral efforts. Perceived costs and benefits of participation in a collaborative effort is often related to level of participant involvement. Sustainability planning is characterized both in terms of planning for financial viability over the life of the collaborative effort and the establishment of independently sustainable programs. Community support is defined as strong community relations that support program implementation and avoid resistance to collaborative goals.

In this case study of the CWG experience, we attempted to answer the following questions, which correspond to the domains of internal coalition functioning noted above:

1. How do current and past CWG members characterize the effectiveness, productivity, collaboration, and leadership of the CWG, and what factors do they identify that facilitate or hinder group function (domains of leadership, task focus, and community support)?
2. What are the institutional or individual reasons for participating and the length of their participation in the CWG (domains of interpersonal relationships, task focus, and participant benefit and costs)?
3. What factors appear to contribute to more effective functioning of a global partnership, and what transferable lessons, if any, can be drawn from the collaboration experience of the CWG that can be relevant for future global collaborative partnerships (domains of sustainability planning and community support)?

### Data Collection and Analysis

We conducted this case study using a mixed-methods approach that included in-depth, semi-structured individual interviews with members; a review of key guiding documents such as the CWG Terms of Reference and externally facing descriptions of the CWG's scope and purpose; and participant observation by 2 of the authors who were active members in the group. An independent consultant was engaged to develop and refine the questions, conduct the interviews, and analyze the findings. In total, the consultant interviewed 19 current and past members of the CWG—out of 21 originally identified—over a 6-month period. Nine open-ended questions were formulated, pretested, revised, and reordered based on the pretest results.

With respondent consent, interviews were digitally recorded and reviewed for clarity and accuracy. Text from the responses was entered in a Microsoft Excel spreadsheet by respondent name and affiliation and by question, and then coded. Key commonalities were identified and consolidated into categories and themes for each domain. The authors also conducted a retrospective analysis of key events that shaped the evolution of the CWG and global scale of the chlorhexidine intervention.

### Ethical Approval

The PATH Research Determination Committee (RDC) reviewed this activity and determined it is not human subjects research as it does not meet the definition of research provided by the U.S. government [45 CFR 46.102(f)] and the Centers for Disease Control. Respondents consented to participate in the interview prior to scheduling the interview and again at the time of the interview prior to answering any questions. The interviewer explained the purpose of the case study, how long the interview would take, and how the results would be used. Respondents were able to refuse to answer any question and/or discontinue the interview at any time.

## FINDINGS

The Table describes respondent affiliations and when they joined the CWG. Of the 19 interviewees, 9 were affiliated with international NGOs, 4 were from pharmaceutical companies, 1 was from academia, 3 were from either bilateral or foundation donors, and 2 represented UN agencies. Professional backgrounds included pediatrics and medicine; maternal and child health program implementation; epidemiology; social science research; public health advocacy; product development, manufacturing, commercialization, and introduction; and global and national policy and advocacy. Seven of the 19 respondents were based in a developing country and the others were based in either the United States or Europe. Just over half the respondents were part of the CWG from its early, more informal start.

**TABLE.** Respondent Profiles, by Year of Joining the Chlorhexidine Working Group^a^

Affiliation	Joined Between 2002 and 2011	Joined in 2012 or Afterward	Total
NGOs	6	3	9
Donors	2	1	3
Academia	1	0	1
Pharmaceutical	1	3	4
United Nations agencies	0	2	2
Total	10	9	19

a The working group was formalized in 2012, but ad hoc collaboration had been ongoing since 2002.

### Background of the CWG

**FIGURE 2.**
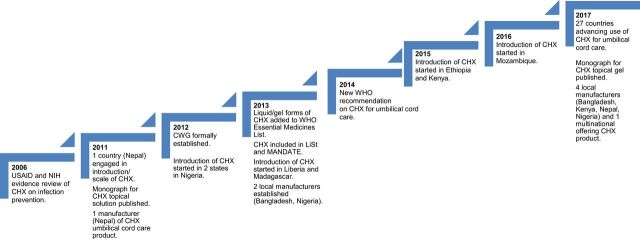
Timeline of Key Milestones Related to the Chlorhexidine Working Group, 2006–2017 Abbreviations: CHX, chlorhexidine; CWG, Chlorhexidine Working Group; LiST, Lives Saved Tool; MANDATE, Maternal and Neonatal Directed Assessment of Technologies; NIH, National Institutes of Health; USAID, United States Agency for International Development; WHO, World Health Organization.

Between 2002 and 2005, chlorhexidine digluconate (7.1% chlorhexidine digluconate, which delivers 4% free chlorhexidine) was evaluated for umbilical cord care for the first time in a large community-based cluster randomized controlled trial in Nepal (Figure 2). The study, published in 2006,^13^ showed a 75% reduction in severe cord infection in the chlorhexidine clusters compared with the dry cord care group and 34% fewer deaths among infants receiving the intervention within the first 24 hours compared with the control arm. These findings drew marked interested and prompted several replication trials.

Between 2002 and 2012, the CWG operated on an intermittent, informal basis with a small core group of interested individuals maintaining momentum for further efficacy studies, implementation research, global advocacy, and programmatic effort. Like the more formalized CWG that developed later, these individuals represented international NGOs, donors (primarily the United States Agency for International Development [USAID] and the Bill & Melinda Gates Foundation), academia, UN agencies, and pharmaceutical companies, although the diversity and breadth of membership was more limited. In 2005, after completion of the Nepal trial but before the results were published, USAID convened a group of neonatal health experts and researchers in Washington, DC to review the results and other evidence for the efficacy and safety of chlorhexidine to prevent newborn infection in low-income countries and to discuss programmatic implications of the findings.^14^ The group determined that, although certainly further replication trials were needed to confirm effectiveness, an accelerated effort to prepare for introduction of chlorhexidine should be undertaken in parallel (Box). The group outlined a multipronged approach to move forward simultaneously with research and program and product planning. This meeting catalyzed momentum around applying an accelerated research-to-use process, conceived by USAID, to chlorhexidine for umbilical cord care in low- and middle-income countries, and it established USAID in a prominent role as funder and visionary of this effort. By 2012, 2 additional randomized controlled trials in Bangladesh and Pakistan had demonstrated the efficacy of chlorhexidine for reducing risk of cord infections and newborn deaths.^15^^,^^16^ A subsequent meta-analysis found a 23% reduction in risk of death.^17^

Between 2002 and 2012, the Chlorhexidine Working Group operated on an intermittent, informal basis.

The working group applied an accelerated research-to-use process to chlorhexidine for umbilical cord care.

BOX **Characteristics of Chlorhexidine for Cord Care Conducive to Wide Uptake**

Everett Rogers^19^ has drawn attention to the characteristics of an innovation that can influence probability of widespread uptake or diffusion, notably: relative advantage compared to current practice; compatibility with existing values, needs, etc. (also can be understood as “acceptability”); complexity/simplicity (with regard to understandability and use); trialability (i.e., feasibility of initially using on a limited basis); and observability of results or benefit. As intervention or innovation characteristics that favor effective delivery at scale, Shelton^20^ adds: how significant a population health burden the innovation could avert; cost; individual efficacy; compatibility with provider attitudes, medical culture, and organization of work; ease of integration within current practices or services; regulatory and policy barriers or facilitators; logistical requirements; commercial-sector compatibility; and single versus multiple benefits. On most of these counts, conditions have been relatively favorable for widespread uptake of chlorhexidine for umbilical cord care. Specific characteristics of the innovation that are conducive to wide uptake include:

- This antiseptic is relatively inexpensive.
- It has long been used in health care, particularly for skin asepsis.
- In many country settings, families and providers conform to an established practice of using substances on the umbilical cord to protect the infant.
- Dramatic early data around the lifesaving potential made the need for the product easily understood and helped create an emotional connection to the intervention.
- The application is straightforward and easy to understand, and it requires minimal training.
- The intervention can be easily integrated into existing essential maternity care and newborn care programming.
- Manufacturing requirements of the product are amenable for local production.

There have, however, been certain innovation characteristics that have created challenges in achieving wider uptake, notably:

- Ambiguity in the evidence base with regard to the most effective application regimen (1 versus 7 days) as well as conditions under which a mortality reduction benefit could be expected. There has been some variability in how partners have dealt with this ambiguity and, at times, this has undermined collaboration effectiveness.
- Lack of a conventional drug development process meant that dose-response data were not available to assist in determination of the exact drug concentration in the product, which means that it is difficult to explain the rationale for the 7.1% level of active ingredient.
- Lack of comparative efficacy data with alcohol, which is used customarily in many countries at facility and home to cleanse the cord after cutting.

The advent of the United Nations Commission on Life-Saving Commodities for Women and Children (UNCoLSC)^18^ in 2012 transformed an ad hoc group of individuals and agencies interested in advancing use of the chlorhexidine intervention into a formalized component of a global effort to expand availability and access to 13 lifesaving commodities. Funding from the UNCoLSC and USAID supported the establishment of a chlorhexidine secretariat and mandated the group as a technical resource team under the auspices of the UN effort. The CWG Secretariat, housed at PATH, an international NGO focusing on global health, sought to encourage a participatory leadership model to support introduction and scale up of chlorhexidine for umbilical cord care. Functioning as a global uptake coordinator and market manager, the CWG has worked across the domains of policy advocacy, coordination, knowledge management, and technical assistance, with participation from more than 30 civil society organizations, universities, ministries of health, pharmaceutical manufacturers, and multilateral organizations. Its composition has brought together individuals and agencies with expertise in product development, manufacturing, supply chain, policy, regulatory requirements, program design and implementation, quality assurance, training, demand creation, behavior change communication, and monitoring and evaluation. The global reach and the collective expertise of the CWG members and their institutions have helped enable the CWG to offer a broad range of tailored technical assistance, in response to requests from country-level partners.

Funding from the UN and USAID formalized establishment of the working group in 2012.

The CWG has been singled out repeatedly within the global maternal-newborn health community as an exemplary case of a collaborative effort that has accelerated scale up of a new intervention.^21^ In order to understand how these outcomes have been achieved, we categorized findings on factors influencing the performance of the CWG across the 6 domains of internal functioning of coalition effectiveness described earlier.

The Chlorhexidine Working Group has been singled out within the maternal-newborn health community as an exemplary case of a collaborative effort that has accelerated scale up of a new intervention.

### Leadership

Overall, respondents felt that—in general—the CWG provided strong leadership with genuinely shared ownership. An important theme emerging from the interviews was the value of technically strong, accountable leadership coupled with transparency and openness, leading to a sense of shared mission and ownership. Particularly, respondents emphasized the benefit of having a neutral “honest broker,” serving in a secretariat role for the working group, creating conditions for collaboration rather than competition. Said one respondent from an NGO:

*The CWG takes into consideration everyone's contribution—everyone contributes. There is no sense of competition. If a country expresses an interest, the CWG provides information on who is working in that country. This is very useful and makes everyone more open to sharing information and helping each other in implementation.*

Such an approach allowed all organizations to share leadership equitably, thus creating a genuine noncompetitive, collegial environment. While the organization responsible for the secretariat function (PATH) also had a specific *technical* role as a *member* of the CWG, it managed to keep this member role separate from its “honest broker” *facilitating* role in support of the operation of the CWG, which was deemed essential to the effective functioning of this partnership. According to an NGO staff person:

*The terms of reference really set the stage for how open the working group would be. We [the Secretariat] really encouraged participation and broad ownership.*

Other words used to describe the functioning of the secretariat included “openness,” “transparency,” “dedication,” “trustworthiness,” “reliability,” and “integrity.” In this regard, PATH, as the convener of the secretariat, formally managed and supported the CWG while at the same time, as a member, shared technical leadership with the various member organizations.

Strong, transparent leadership led to a sense of shared mission and ownership.

Both during the period before 2012, when the group operated on a less formal basis, and later, when supported by a more formal secretariat, almost every member/organization was considered a leader in some specific aspect of the overall effort. An atmosphere of trust and respect that allowed for open, clear, reliable, and timely communication proved critical to support the investment of time, energy, and resources by group members. When funding allowed, biweekly CWG teleconferences and quarterly face-to-face meetings were convened and facilitated by the secretariat. These meetings offered an opportunity for members to participate directly by presenting their current work, updating their peers on new initiatives, and discussing any issues related to product supply and/or implementation. The meetings also provided opportunities to disseminate lessons, identify and facilitate synergies between partners, and advocate for and collaborate on the acceleration of chlorhexidine introduction and scale up. Direct participation, particularly in the face-to-face meetings, offered group members a way to strengthen relationships and build trust, even though using valuable meeting time to report out what could be read is often not considered a good use of meeting time.^22^ Shared leadership was also seen in ongoing informal collaboration, with members in regular communication by phone or electronically, partnering on specific tasks.

#### Interpersonal Relationships

As the operations of the CWG became more formalized, clear terms of reference were developed, which helped the group coalesce around defined goals and targets. The group produced governance documents and made them publicly available. The CWG Terms of Reference, jointly created by its membership, clearly delineated purpose, membership, structure, and objectives, with explicit statements on the importance of transparent collaboration and on expectations for its members.^23^ The accompanying Strategy Statement described the health need being addressed, as well as the CWG's vision, purpose, strategic goals, values, and leadership. The Capacity Statement summarized the CWG's intent, activities, membership, and available resources. Taken collectively, these documents covered a full range of governance, strategy, and purpose issues and provided a framework for CWG functioning.

Clear terms of reference helped the group coalesce around defined goals and targets.

All participants were invited to attend face-to-face meetings and regular teleconferences and were encouraged to use the information they obtained through these communications to achieve wider availability, accessibility, and affordability of the product. Country point people updated the group regularly about progress and/or barriers to introduction at the country level and were responsible for representing the CWG to national stakeholders, which contributed to the sense that each member was a valued part of a bigger effort. This served to complement their ongoing organizational effort and fostered collaborative rather than competitive norms. Further, this type of participatory leadership model helped to assure CWG members that the secretariat was a trusted partner that willingly shared both financial resources and technical credit with all members.

Good interpersonal communication engendered a sense of collegiality, which was evident in the numerous side interactions that took place among CWG members outside of formal meetings. Members communicated and collaborated beyond the formal meetings in an intentional yet informal way, whether about research, policy, supply, or other issues. These less formal interactions were pivotal in that most key decisions beyond a single organization appeared to be made during these interactions, rather than in the larger, more-structured meetings.

Most key decisions beyond a single organization appeared to be made during less formal interactions than in structured meetings.

#### Task Focus

Respondents felt that an important contributor to the effectiveness of the CWG was that it has had a clear mission and mandate. Critically, the group jointly developed shared goals and specific actions to be undertaken at the global and country levels. Through regular teleconferences and face-to-face meetings, these shared goals and actions were jointly reviewed, helping to ensure group accountability. The goal was always to draw on the collective expertise of the multidisciplinary CWG membership to advance introduction, scale up, and effective delivery of chlorhexidine.

Several of those interviewed pointed to the role of the CWG as an “uptake coordinator,” by setting targets, defining strategies globally, and providing technical assistance to country programs, all aided by systematic sharing of information. Respondents felt this was a productive model for future collaborative partnerships. One donor referred to this function as analogous to that of a “product manager”:

*Much of what limits scale up is a lack of transparency and visibility about what is going on [with a product]. While there was no formal accountability within the CWG, it served as a sort of product manager. An uptake coordinator is ultimately accountable for global scale-up (e.g., not just in the first few countries). The uptake coordinator needs to see the big picture and should be granted the authority to lead, make decisions, and influence outcomes.”*

The CWG was facilitated in its role as uptake coordinator by having, as secretariat, an organization that was seen as playing an unbiased role (in this case, PATH). An organization playing this role must be trusted by all relevant stakeholders including ministry of health representatives. This respondent went on to say that having a clear mandate and adhering to rigorous project management standards helped provide the group with credibility, which, in turn, helped attract new members, who felt reassured that their involvement would be worthwhile and that the technical rigor of the group would meet their standards.

Several respondents noted that a helpful attribute of CWG meetings has been that they have been tightly organized, which kept the group task-focused and efficient. Telephone and in-person meetings were preceded by clear agenda items and focused on action; minutes were uniformly shared; and face-to-face meetings were convened periodically to review new evidence and report on product introduction progress. The clear, reliable structure of communication, documentation, and convening “fostered a sense of being part of a joint effort, part of a community,” said an NGO representative.

Tightly organized meetings kept the group task-focused and efficient.

The CWG functioned as both an advocacy and technical assistance resource. Some group members felt that the advocacy and evidence-synthesis functions were sometimes pulling in different directions. In this case, the twin focus of the CWG meant, to some people, that chlorhexidine was being advanced in any country that was interested, without sufficient attention to existing data.

Although target setting was a noted strength of the CWG, several respondents expressed that the group could benefit from improved process and articulation of targets for utilization and tracking progress to ensure accountability. According to 2 respondents, the CWG became more of an advocacy body for the product than an objective source of information and technical assistance, with recommendations for introduction and scale up being perceived as being promoted before the full range of evidence was available. A USAID colleague also noted that:

*With any intervention, the people involved will become extremely invested in advancing it. This is not unusual. Could there have been more introspection about the limitations, but also active debate? … I don't know if we really settled this.*

This suggests that how the CWG has functioned has not always fostered sufficient critical reflection and debate on strategy. This tension around strategy for advancing chlorhexidine led, at times, to a lack of shared task focus, which undermined collaboration.

#### Participant Benefits and Costs

To continue to participate, members must perceive that the benefits of collaborating outweigh the costs (e.g., meeting attendance, time, labor, opportunity costs associated with prioritizing focus on one intervention over another). Respondents noted that a key benefit was that the process fostered effective translation of research to action. Respondents across disciplines noted that the CWG provided a forum for reviewing evidence as it emerged and determining the implications for program implementation. For CWG members residing outside the United States, having access to evidence and technical assistance was essential for making the case for chlorhexidine introduction with national-level stakeholders, including ministries of health and regulatory and policy authorities. Several respondents noted the importance of having technical briefs and research summaries available to share with stakeholders, as well as information on how various countries were approaching product introduction and integration. Access to this information enabled individual group members to provide technical assistance and guidance at the country level, both within their own countries as well as in others. A Nepal-based NGO representative noted that:

*Without this forum, there would be no scale up. We used it to share our experience, which then allowed us to reach so many other countries. We have now provided technical assistance to eight other countries.*

Evidence was also used to develop specifications for manufacturing and product development and support regulatory and licensing processes. For the pharmaceutical company members of the group, having access to evidence on product effectiveness and program implementation experience, as well as access to technical assistance on manufacturing and technology transfer requirements, helped them plan for product introduction and ensure product quality. The openness with which information was shared also encouraged even potentially competitive entities to share experiences so that all members could learn from both successes and challenges that individual programs or countries were experiencing. According to an NGO representative, this openness, combined with project management rigor and reliability, “increased the value of the group in the eyes of many people beyond the small group that initiated this.”

The CWG appeared to be relevant to multiple, diverse stakeholders—as reflected in the CWG's technical and geographic diversity. Numerous respondents commented on the value of having members from a broad range of disciplines, technical expertise, and geographic diversity. This collective expertise enabled the CWG to provide guidance and assistance throughout the product development and introduction value chain—from product formulation to ultimate delivery. This design was deliberate, according to a USAID respondent, who had been involved from the early days.

*This was very much an explicit effort … to bring together a diverse group to think about how we roll this out. How can we be smarter? Can we anticipate what challenges we would encounter?*

Another donor representative stated, however, that:

*[although it was] definitely important that the CWG existed, it also felt like its [work] could have been considerably more time-efficient and cost-effective … incentives were not always aligned for action.*

As we have noted, the CWG leadership made explicit efforts to encourage a collaborative, inclusive, and supportive approach, drawing on a technically and geographically diverse membership. A governing tenet was to be all-inclusive by offering membership to any interested party, including industry. Some tensions arose initially with the inclusion of pharmaceutical companies, with the perception of favoritism or providing commercial advantage needing to be managed carefully. Assisting the group members to ascertain an optimal role for their engagement increased collaboration effectiveness. Early on, members reached out directly to engage various multinational pharmaceutical companies in the production and distribution of chlorhexidine. This was somewhat at odds with the stated intention of the group to build local/regional production capacity^24^ and could have reflected a lack of clear articulation and/or understanding of shared goals among group members. The multinational pharmaceutical company that chose to remain engaged in the CWG supported this strategy and communicated its role as a “back-up supplier” very clearly to the group.

Generally, pharmaceutical companies are more interested in market share than program implementers, who have more of a public health vision. Commercial interests were not addressed directly, even though for-profit pharmaceutical company representatives were active members of the group. Instead, all members were advised about the lack of confidentiality of information being shared and encouraged to use their own discretion to decide what information to share, considering prior and existing agreements with other organizations. One pharmaceutical company representative noted that:

*The group didn't talk about commercial interests at all. This was left to the companies to figure out. And there was not conflict of interest because our risk was different, our regions of operation were different.*

CWG members operate in a competitive environment, both in the pharmaceutical company and technical assistance spheres. Often these members are bidding for the same business (i.e., product orders, donor-funded scopes of work) so the potential for obtaining an unfair competitive advantage through group membership is real. To address this, the CWG maintained a strict commitment to transparency by sharing all materials and information with all members of the group to avoid putting any specific members at a disadvantage. This normative behavior built trust among group members and reduced overt competitive behavior.

While some participants have been actively involved in chlorhexidine-related program implementation, the CWG also has welcomed organizations expressing an interest in participating in the CWG on a more passive basis to obtain or provide certain information. Several respondents pointed to the group's openness to having new members join along the way as a key strength given that critical challenges and issues changed over time, requiring different expertise. This flexibility enabled the working group to respond to emerging needs while maintaining a clear focus on the overall goal. The CWG's responsiveness, especially to inquiries from the country level, was noted repeatedly, along with the value of having available online evidence synthesis, technical, and advocacy materials.

Ad hoc, time-limited subgroups were also created within the CWG when in-depth work on specific issues was needed. This included provision of country-specific guidance and support to country initiatives, support on local/regional manufacturer production and related market analysis, the development and implementation of advocacy and dissemination strategies, and creation of monitoring and evaluation indicators.

Finally, respondents valued the group's ability to deal with a wide range of technical issues because of its diversity. The mix of viewpoints and expertise enabled the group to think proactively from an integrated health systems perspective about the approach to scale-up in a particular country.

Early on, country point people were identified from organizations that were active or had the potential to be active in introducing and scaling up chlorhexidine in a given country. This country-level leadership was spread across many CWG members, which built shared commitment and accountability at the country level. When a country showed initial interest in the intervention, the CWG would discuss and determine jointly the best person or organization to liaise with national stakeholders. In some circumstances, this support included using funds allocated to the CWG Secretariat to support CWG members to visit countries to conduct initial technical consultations and provide short-term technical assistance. These technical assistance visits benefited not only the country but also the point people and their organizations as they were able to be called upon by national leadership for longer-term technical assistance for introduction and scale planning.

#### Sustainability Planning

The CWG received funding for a formal secretariat in 2012, 10 years after its emergence as an ad hoc interest group. This support was critical to the functioning of the secretariat, provision of in-country technical assistance, development and maintenance of key informational materials, and arrangement of face-to-face meetings.

Funding support was critical to the functioning of the secretariat, provision of in-country technical assistance, and development of key materials.

Activities of the CWG model have not been formally costed. The level of effort expended by PATH to support the working group, specifically for facilitating and managing the secretariat and hosting meetings (excluding PATH's technical work on chlorhexidine), was the equivalent of 1.5 full-time staff positions. One full-time person, with master of public health (MPH) training, managed the daily tasks and 2 other more senior staff contributed about one-quarter time each for overall leadership and technical input. Budget elements supporting the CWG included limited venue costs to host periodic face-to-face meetings, printing costs for selected CWG materials, communication, and travel to attend relevant meetings. Member organizations in the CWG covered their own travel costs and time to attend the face-to-face meetings.

The CWG has nurtured the establishment of independently sustainable programs as well. Since 2011, the CWG has supported planning for introduction of chlorhexidine for umbilical cord care in more than 25 countries. Chlorhexidine is now produced by local manufacturers in Bangladesh, Kenya, Nepal, and Nigeria. Through 2016, approximately 5.5 million doses of chlorhexidine have been distributed by these manufacturers. The United Nations Children's Fund (UNICEF) also distributed approximately 2.7 million doses up to 2015.

Of those interviewed, 6 respondents expressed that the CWG was instrumental in accelerating national adoption and scale up of chlorhexidine for umbilical cord care, in terms of both number of countries and number of users. An NGO respondent stated that:

*Without this working group, we would not be close to where we are in introducing chlorhexidine in countries.*

Similarly, an NGO staffer based in Nigeria noted that scale up was greatly facilitated by the group having guidance materials available and “having a network of support” that facilitated access to key resources, evidence, and technical expertise not otherwise typically available. Several respondents remarked on the strategy used by the working group of simultaneously supporting generation of additional evidence on efficacy and priming the product for introduction by identifying potential local manufacturers and addressing potential policy changes. This approach, contrasted with a more linear, sequential model, was not supported by all members at the start but was viewed by most in retrospect as a more time-efficient approach to product introduction. A respondent from academia explained:

*USAID wanted to have efforts ongoing even while evidence was being generated. The idea was to reduce the time frame between evidence generation and going to scale. By having a group that can engage at different points in the process, we could problem solve and apply lessons learned.*

Participation of pharmaceutical companies as members in the CWG was considered an asset by many members. Those from national pharmaceutical companies believed that their participation helped them build capacity. A pharmaceutical company representative based in Nigeria stated that involvement in the working group helped them become “a world-class company” because of what they have been able to learn not just about manufacturing but also about effective advocacy. Said this respondent:

*This is a new product that can change old ways that can be harmful. Changing from old practices to new ones takes a lot of advocacy. We didn't realize this, but we now understand that it is important to work with governments to change practices. We now see ourselves as a world company, not a local company. We are lifted. We also see the potential that lies ahead of us if we continue to do our best.*

#### Community Support

To support the global health community, the CWG created an effective clearinghouse of information for advocacy and implementation. With funding from the UNCoLSC, the CWG was able to establish a web-based comprehensive information platform that provides free access to global and country-level tools and reports hosted by Save the Children's Healthy Newborn Network portal,^25^ which greatly improved access to information. This has meant that implementing partners, manufacturers, ministries of health, and other stakeholders have had readily available information on a range of topics and were not isolated in their efforts. This also conveyed a sense that the CWG was client-focused. As an NGO representative noted, it demonstrated:

*a serious effort to understand what [clients] are facing, what their needs are, and what type of information was important to trying to address those needs.*

This respondent felt that this was essential to the working group ultimately achieving its mandate to catalyze scale up and save lives. Another essential element to providing relevant information was the long-term commitment to updating evidence and resources.

As the CWG expanded over the years, membership became more diverse and representative of the various sectors in the global health community that could be instrumental in program implementation. International bodies such as the World Health Organization (WHO) and UNICEF are pivotal members in the global health community, and close collaboration among these bodies is generally sought. Several respondents commented that greater involvement and support from these bodies could have increased the effectiveness of the CWG. Although UNICEF underwrote some of the costs related to CWG activities as part of UNCoLSC, their engagement was uneven, primarily due to turnover in staff assigned to address newborn health and related staffing needs for global crises such as Ebola. WHO joined the group once it was formalized through UNCoLSC, but tensions arose around balancing the role of WHO as a normative body with the practicalities of creating sufficient demand and supply to make the product available for those most in need. For example, the accelerated process of research-to-use employed by the CWG was perceived to be inconsistent with the formal and more lengthy WHO process for evidence review and formation and approval of new intervention guidelines. Because WHO, a key player in the global community, eventually disengaged from active participation in the CWG, the overall scope of collaboration that the CWG was able to achieve was reduced.

Greater involvement of international bodies such as WHO and UNICEF could have increased effectiveness of the working group.

## DISCUSSION

A secretariat model, in which an organization plays the role of convener, coordinator, and manager of working group activities, appears to be an effective means of support for collaborative partnerships in global health. In the case of the CWG, this structure offered an established platform to manage a diverse range of activities related to scale up of the intervention while at the same time sharing technical leadership across the membership. Creating a successful collaboration with tangible outcomes requires that the organization playing the facilitation or secretariat function be seen as trustworthy by all members. PATH, playing a secretariat role for the CWG, attempted to do so transparently and collaboratively using a participatory leadership approach,^26^^,^^27^ which essentially devolved leadership and encouraged shared ownership among all members.

The organization playing the facilitation function must be seen as trustworthy by all working group members.

Seeking involvement and feedback from members in the development of guiding documents was intentional and helped normalize participation and trust as a member experience. Transparency and flexibility in meeting agenda and structure appeared to incentivize members to share openly. Members received recognition and immediate feedback from their peers, which seemed to foster effective interpersonal communication.

While all members were motivated to participate, and did so without financial support from the secretariat, a global collaborative partnership cannot always be sustained in the absence of dedicated resources. Sufficient available resources over the short term, when critical efforts around global awareness raising and initial country rollout are being implemented, can accelerate progress at both global and country levels.

Currently, due to lack of funding, the formal secretariat is no longer functioning. It is likely that the commitment to advancing chlorhexidine for umbilical cord care among key CWG members will remain and some elements of group function will be sustained on a less formalized, as-needed basis, as in the earlier history of the group. Negative aspects to allocating dedicated funds to institutionalize a group function (e.g., the secretariat) extend to the generally problematic issue of accessing continued funding to support group activities. On the other hand, allocating dedicated funds helped ensure accountability for centralized resource and knowledge management, ensured availability of point persons who were actively scanning and apprising global and country situations, and provided incentives for nurturing and sustaining involvement across a broad cross-section of members.

Closer coordination with normative bodies (in this case, WHO) was cited by respondents as an area to strengthen. More explicit and detailed discussion on the available and pending evidence and its implications for how and where to prioritize introduction of chlorhexidine could have been helpful in bridging this gap. In some cases, however, differences in how groups understand their mandates may not allow the flexibility that close collaboration requires.

Shiffman reviewed 8 case studies of global health networks and identified 4 common strategic challenges: problem identification, positioning of the network, coalition building, and governance.^28^ For the most part, CWG members concurred on problem identification (high newborn mortality) while struggling somewhat with consensus around positioning (chlorhexidine as an effective way to reduce newborn morbidity and mortality) due to new evidence from randomized controlled trials conducted in Africa.^29^^,^^30^ This new evidence failed to show a mortality effect of chlorhexidine in populations with relatively lower neonatal mortality, although—as with the earlier trials—cord infections were reduced. With these new results, some internal and external members questioned the positioning or framing for external audiences of chlorhexidine as an effective way to reduce newborn mortality in *all* settings. Framing chlorhexidine as an intervention that can be expected, necessarily, to reduce mortality risk, has had to be revisited. Instead, greater priority is being given to ensure availability in high-mortality settings and populations and to highlight the benefits of the use of chlorhexidine for reducing risk of cord infections across all populations.^31^

As noted in the USAID publication *Idea to Impact*^32^:

*Global coordination requires effort – something not always recognized in the global health community. Significant resources are needed to manage internal communications and logistics alone. However, deep technical and strategic skills and expertise are needed to support, shape, and prioritize all the activities across all the functional areas of expertise.*

A recent study among African and global health system professionals documented the lack of shared understanding around what and how to realize effective global partnerships.^33^ We believe that the participatory collaborative approach used by the CWG offers an instructive example and insights into the factors that can help or hinder global health collaboration. For example, considerable evidence suggests that successful collaboration in global health is characterized by both discipline and flexibility in management during implementation.^34^ A key lesson from the CWG experience was that, to the extent possible, clear, realistic targets and time frames need to be established, as well as specific criteria for when the work of the partnership is done.

The current case study involved document review and key informant interviews. As both a strength and potential limitation, 2 of the authors have been direct participants in this collaborative effort. This involvement—on the one hand—means that conclusions cannot be considered to be entirely neutral and objective. On the other hand, such a methodology, more systematically tapping the insights of direct participants, can yield deeper learning. This is, indeed, a principle of action research and participatory evaluation.^35^^,^^36^

## CONCLUSION

Effective collaboration in the case of the Chlorhexidine Working Group appeared to be a consequence of: (1) leadership that maintained a careful balance between discipline and flexibility, (2) a strong secretariat structure that supported the evolution of trust and transparent communication in interpersonal relationships, (3) shared goals that allowed for task focus, (4) diverse membership and active involvement from country-level participants, which created a positive benefit-cost ratio for participants, (5) sufficient resources to support the partnership and build sustainable capacity for members to accelerate the transfer of knowledge, and (6) support from the global health community across multiple organizations.

## Notes

**Peer Reviewed**

**Funding:** USAID HealthTech V; UNICEF through the United Nations Commission on Life-Saving Commodities for Women and Children; and the Bill & Melinda Gates Foundation.
